# Analysis of the anomie behavior and external motivation of college students in sports: A cross-sectional study among gender

**DOI:** 10.3389/fpsyg.2022.988557

**Published:** 2022-09-13

**Authors:** Liping Liu, Shanping Chen, Xueyan Yang

**Affiliations:** ^1^School of Public Policy and Administration, Xi’an Jiaotong University, Xi’an, China; ^2^Department of Physical Education, Xi’an Jiaotong University, Xi’an, China

**Keywords:** external motivation, gender, undergraduate, anomie behavior, sports

## Abstract

**Objective:**

In the context of Healthy China, the effect of external motivation on sports anomie behavior from the perspective of gender among Chinese college students was investigated.

**Participants:**

In total, 2,340 college students were involved in this study.

**Methods:**

The self-made scales were used, which were about anomie behavior and external motivation in sports. The independent sample *T*-test was used to compare the sports anomie behavior between male and female students. Then, multiple linear regression analysis was adopted to examine the effects of external motivation on sports anomie behavior.

**Results:**

Girls’ sports anomie behavior was lower than boys. There were significant gender differences in honor motivation, obedience motivation, and economic motivation; girls’ motivations were lower than boys. The academic motivation was negatively correlated with the sports anomie behavior of college students of both genders. The economic motivation was positively correlated with their sports anomie behavior. The honor motivation was negatively correlated with the sports anomie behavior of female college students.

**Conclusion:**

Female college students’ sports anomie behavior and external motivation are lower than that of male college students. External motivation had a significant influence on college students’ sports anomie behavior.

## Introduction

The outline of “Healthy China 2030” clearly proposes that school and health education should be strengthened, and the health literacy of citizens should be improved. It also proposes that the national fitness campaign should be widely implemented and that self-discipline should be promoted to foster healthy behavior. In recent years, the anomie behavior of college students in their sports activities has attracted the attention of scholars, as this has seriously impeded the cultivation of healthy behavior among college students (Chen et al., 2018). Studies have shown that male and female college students had great differences in sports attitudes, motivations, and sports behavior (Liu et al., 2017). Some studies have posited that there are significant differences in the sports behavior of male and female college students, specifically in their thoughts, consciousness, and self-exercise habits. Given all of this, determining how to foster healthy sports behavior among male and female college students and reduce sports anomie is an urgent task.

Anomie behavior, which is also known as deviant behavior and differential behavior, is proposed by Durkheim and refers to behavior that deviates from the norms that members of society abide by. In Durkheim’s view, anomie is a kind of irregularity or lack of social norms and confusion (Anthony and Philip, 2019). In essence, it is when behavior violates existing standards and norms of a society or group that relate to morality and law. It is when behavior deviates from the established “normal operation track” (Zhou, 2011). According to Douglas and Waxler (1987), deviant behavior refers to a subject’s behavior when he/she deviates from the established track or to the behavior of social members when they deviate or violate norms to varying degrees. Deviant behavior is also called “improper behavior” or “differential behavior.” According to sociology, there are five levels of deviant behavior in sports, namely, behavior against customs, behavior against discipline, behavior against morality, illegal behavior, and criminal behavior (Luo and Zhou, 2011). Zhang and Wang (2021) proposed in their research that sports anomie refers to sports participation behavior in which the subject violates laws, ethics, and rules in the process of sports participation, and sports anomie behavior and compliance behavior can be regarded as a mirror of behavioral norms front and back sides. Following this classification, this study defined the sports anomie behavior of college students as the behavior wherein college students violate social norms in sports, such as morality, rules and regulations, competition rules and laws, and regulations in activities.

During the peak period of physical and mental development, adolescents are highly curious and inquiring, but have poor self-control and low self-discipline, and are prone to anomie behavior (Liu and Shi, 2016). From the analysis of existing literature, scholars have already conducted research on the behavior of athletes, coaches, and other sports workers in competitive sports (Zhou, 2021). A small amount of research has been carried out on the phenomenon of negative sports behavior, while the research on sports behavior of college students mainly focuses on the positive impact of sports on physical and mental health. Quantitative research, its formation mechanism, and intervention methods are still theoretical gaps.

Based on the behavioral motivation research of SDT, exercise motivation has a psychological orientation effect on sports persistence (Edmunds et al., 2006; Ma, 2014). Good motivation can drive students to take the initiative to participate in sports activities and then develop the habit of exercise (Chen and Li, 2007). Motivation is divided into internal motivation and external motivation. Psychology defines internal motivation as participation in an activity for the satisfaction of the behavior itself, and external motivation is participating in activities due to external pressure, rewards, and returns (Richard and Ryan, 2000). Existing internal motivation measures include five dimensions, namely, health motivation, appearance motivation, fun motivation, ability motivation, and social motivation; extrinsic motivation measures include two dimensions, namely, academic motivation and obedience motivation. The needs involved in internal motivation can only be met by normal exercise behavior itself. It has been proved that internal motivation and sports behavior have a positive correlation, while external motivation and sports behavior have a negative correlation (Shang, 2017). With the development of the market economy, the values of sports people are also quietly changing, and people’s pursuit of money and honor is getting stronger and stronger, causing some athletes to lose their sportsmanship and obtain competition rankings through various improper means, seek the satisfaction of vanity (Pan, 2019). From the perspective of behavior motivational conflict, individuals intend to resist external temptations before making a behavioral decision (Arkinson and Birch, 1970; Gollwitzer et al., 2004). However, this intention depends on the difficulty of the motivational goal (Kruglanski et al., 2002). Needs related to external motivation may be met by exercising the above-described normal behavior or others. When these external needs cannot be met by normal sports behavior (or when it is difficult or inconvenient to implement the normal behavior), the needs of the score and award are likely to be met by improper means (Ryan and Deci, 2000). These improper means are against the norms of sports behavior and as such may be classified as anomie behavior. Therefore, the external motivation to exercise may lead to sports anomie behavior (Chen and Li, 2007). Based on the data of Chinese college students, this study analyzes the differences between male and female college students’ exercise motivation and the influence of sports anomie behavior, which shows a certain theoretical and practical significance for China and other countries to study students’ sports behavior and motivation theory.

In the existing research on physical exercise behavior, not only the demography factor is used as a regulating variable to investigate physical exercise behavior but also the psychological variable is used as a factor to explain behavior occurrence (Miao and Qin, 2006; Taymoor and Lubans, 2008). Existing studies have incorporated individual characteristic variables, such as age, an only child, and a cadre of the class and group into the research fields, and believe that these characteristic variables will also affect college students’ sports behavior and psychological factors (Yan et al., 2016; Chen, 2022).

Consequently, the purpose of this study was to analyze the sports anomie behavior, the external motivation, and its influencing factors of male and female college students to improve their behavior regarding sports health. It proposes strategies to specifically intervene in the external motivation of college students to exercise, standardize, and prevent sports anomie behavior of male and female students to promote healthy sports behavior of male and female college students.

## Materials and methods

### Data collection and sample

This research is divided into three phases. The first phase of qualitative research uses stratified random sampling to select 63 college students from 3 universities in Northwestern China for qualitative interviews and related theoretical research, using Likert’s 5-level scale to design measurement questions for exercise external motivation and sports anomie behavior items. In the second phase of the pre-survey phase, stratified random sampling from 10 May to 30 July 2018, 360 copies were distributed to 3 colleges and universities in the northwest region, and 338 valid samples were recovered with an effective rate of 93.8%. The internal consistency of this scale was found to be adequate for this study. In the third phase, a national formal questionnaire survey was conducted. From 1 October 2018 to 19 December 2019, 12 universities (5 in North China, 2 in Northeast China, and 5 in East China) and 8 colleges (3 colleges in central and southern regions, 3 colleges in southwest regions, and 2 colleges in northwest regions) were selected by stratified random sampling. The college students were issued 2,400 questionnaires (120 questionnaires per college, 30 questionnaires for each grade, and 15 for men and women), and 2,340 valid questionnaires were returned. The response rate reached 97.5%.

The research objects are college students. The 2,340 valid questionnaires included 1,145 females and 1,195 males; 1,187 undergraduates from key colleges and universities, 1,153 undergraduates from ordinary colleges and universities; 598 first-year college students, 603 second-year college students, 572 third-year college students, 567 fourth-year college students; 975 only child students, 1,364 non-only child students; 936 class leaders, 1,404 non-class leaders (see Table 1).

**TABLE 1:** Descriptive statistics of sample characteristics (*n* = 2,340).

Variable name	Code/definition	Percent/mean (SD)
Gender	0 = female; 1 = male	51.07%; 48.93%
School level	0 = ordinary colleges and universities; 1 = key colleges and universities	49.27%; 50.73%
Grade	1 = first grade; 2 = second grade; 3 = third grade; 4 = fourth grade	25.56%; 25.77%; 24.44%; 24.23%
Is it an only child	0 = non-only child; 1 = only child	58.32%; 41.68%
Is it a class leader	0 = non-class leaders; 1 = class leader	60.00%; 40.00%
Sports anomie	1 = never; 2 = rarely; 3 = sometimes; 4 = often; 5 = always	1.12 (0.24)
Academic motivation	1 = no; 2 = a little; 3 = strong; 4 = stronger; 5 = very strong	4.44 (0.80)
Obey motivation	1 = no; 2 = a little; 3 = strong; 4 = stronger; 5 = very strong	3.83 (1.15)
Economic motivation	1 = no; 2 = a little; 3 = strong; 4 = stronger; 5 = very strong	3.28 (1.42)
Honor motivation	1 = no; 2 = a little; 3 = strong; 4 = stronger; 5 = very strong	4.04 (1.05)

### Measurement

At present, there is no mature measurement tool for college students’ sports anomie behavior. According to the five major sports activities of college students, namely, physical education class, National Physical Health Standard Test for Students (hereinafter referred to as the Standard), sports competition, sports associations, and independent exercise, combined with the interview with students, a measurement scale with 55 questions was compiled by the research team. This included 1–16 questions regarding physical education class, such as “late in physical education class” and “early leave.” Regarding Standard, there were 17–29 questions, such as “cheating.” Regarding sports competition, there were 30–40 questions, such as “intentional foul in extracurricular sports competition.” Regarding sports association, there were 41–49 questions, such as “failing to complete related tasks in sports association” and “shirking responsibility.” Regarding self-exercise, there were 50–55 questions, such as “intentionally damaging sports equipment and facilities in extracurricular independent exercise.” The number of times this kind of behavior happened was asked. The mean value of 55 questions indicated the score of sports anomie behavior, which had a range from 1 to 5 representing “never,” “rarely,” “sometimes,” “often,” and “always,” respectively (for the value assignment method, see Table 1). This article used Cronbach’s coefficient to test the reliability of the scale. Reliability analysis results showed that the Cronbach’s coefficients of the five subscales of sports anomie behavior were 0.726, 0.695, 0.726, 0.745, and 0.574, and most of them were above 0.7. Although two were lower, they were also close to 0.7, indicating that the questionnaire had a good reliability level.

The external motivation scale is revised based on the existing external motivation scale (Chen et al., 2008). Existing external motivation includes two dimensions of academic motivation and obedience motivation, namely, “I want to meet the attendance requirements of sports activities,” “I want to pass the physical education exam,” “I want to get sport-related scores,” and “I hear the opinions of my friends.” Opinions, “I want to hear the opinions of my classmates” and “I want to hear the opinions of my family.” Based on the theory of external motivation and combined with the interview with college students, two dimensions of economic motivation and honor motivation were added based on the original score motivation and obedience motivation, namely, “I want to meet the attendance requirements of sports activities,” “I want to pass the sports examination,” “I want to get sport-related scores,” “I want to listen to friends’ opinions,” “I want to listen to classmates’ opinions,” and “I want to listen to family members’ opinions.” Second, economic motivation included “I want to use sports activities to get scholarships,” “I want to use sports activities to get some economic benefits,” and “I want to get excellent, research and exchange opportunities.” Finally, under honor motivation, three items included, namely, “I want to win in the competition,” “I want our team to win,” and “I want to get other people’s praise in sports activities.” The score ranged from 1 to 5, indicating “no,” “a little,” “strong,” “stronger,” and “very strong,” respectively. The average value of three questions indicated the score of one dimension. A higher score represented higher motivation in the individual (for the value assignment method, see Table 1). This article used Cronbach’s α coefficient to test the reliability of the scale. Reliability analysis showed that the Cronbach’s α coefficients of academic motivation, obedience motivation, economic motivation, and honor motivation of sports anomie behavior motivation were 0.918, 0.955, 0.879, and 0.880, respectively, which were all above 0.7, indicating that the questionnaire had a good level of reliability.

### Statistical analysis

Statistical data were processed by SPSS 22.0 programs. First, the independent sample *T*-test was adopted to compare and analyze the sports anomie behavior and external motivations of college students of different genders domestically. On this basis, the sports anomie behavior was selected as the dependent variable, and a multiple linear regression analysis method was adopted to investigate the impacting factors of sports anomie behavior between different genders. Among them, Model 1 and Model 1*(1′) take four external motivations (academic motivation, obedience motivation, economic motivation, and honor motivation) as independent variables; in model 2 and Model 2*, school ranking, grade, only-child status, and class leader status were added as control variables.

## Results

### Descriptive statistics

Table 2 showed the analysis results of gender differences in sports anomie behavior and the external motivation scores of college students in China. The results of the independent sample *T*-test showed that in addition to academic motivation, there were also significant gender differences in sports anomie behavior (*P* < 0.001), obedience motivation (*P* < 0.01), economic motivation (*P* < 0.05), and honor motivation (*P* < 0.001). The score of the sports anomie of boys was 1.150, while that of girls was 1.101. The obedience motivation score was 3.908, while that of girls was 3.760. These indicated that the sports anomie and obedience motivation of girls were significantly lower than that of boys, thus showing that girls were less affected by their friends, classmates and family in sports activities than boys were. Furthermore, the score of economic motivation of boys was 3.342, while that of girls was 3.220. The honor motivation score of boys was 4.180, while that of girls was 3.901. These showed that the economic motivation and honor motivation of girls were significantly lower than that of boys.

**TABLE 2:** Descriptive statistics.

Variables	Male (*N* = 1,145)		Female (*N* = 1,195)		*T*
			
	Mean value	SD	Mean value	SD	
Sports anomie	1.150	0.278	1.101	0.185	−4.961***
Academic motivation	4.423	0.818	4.460	0.775	1.114
Obedience motivation	3.908	1.114	3.760	1.179	−3.140**
Economic motivation	3.342	1.445	3.220	1.390	−2.091*
Honor motivation	4.180	0.988	3.901	1.090	−6.483***

*P < 0.1, **P < 0.05, ***P < 0.001.

### Analysis of influencing factors

Table 3 showed the regression analysis results of the influencing factors of sports anomie behavior of male college students in China. In Model 1, obedience motivation and honor motivation had no significant effect on the sports anomie behavior of male college students, but academic motivation and economic motivation did. Among them, academic motivation and sports anomie had a negative significant impact. To meet the requirements of physical attendance, pass the physical examination, and obtain physical credits, stronger academic motivation of male college students was needed because then they would be less likely to adopt anomie behavior (−0.211, *P* < 0.001). Economic motivation and sports anomie had a positive significant impact. However, the stronger motivation of male college students to obtain scholarships and economic benefits meant that they were more likely to adopt anomie behavior (0.071, *P* < 0.1). Following Model 1, Model 2 further included school level, grade, whether the individual was an only child and class cadres as control variables. Then, it was seen that the influence of academic motivation on sports anomie behavior of college students became significant and that the direction of the regression coefficient did not change, but the regression coefficient itself slightly decreased (−0.200, *P* < 0.001). The same thing was seen for economic motivation (0.064, *P* < 0.1). Furthermore, it was seen the hat school level had a significant negative impact on the anomie behavior of male college students. This indicated that the higher the school level, the less the anomie behavior among male college students, which further indicated that grades had a significant positive impact on their anomie behavior. With the increase in grade, there would be a consequent significant increase in the anomie behavior of male college students in junior and senior years. Other control variables had no significant effect on the anomie behavior of male college students. Model 1 and Model 2 were significant; *R*^2^ increased with the increase of variables, which indicated that the model setting was effective.

**TABLE 3:** Multiple linear regression of sports anomie behavior of male college students.

Dependent variable: sports anomie	Model 1	Model 2
Independent variables		
Academic motivation	−0.211***	−0.200***
Obedience motivation	–0.032	0.036
Economic motivation	0.071*	0.064*
Honor motivation	–0.021	–0.027
Control variables		
School level		−0.056*
Sophomore		0.057
Junior		0.076*
Senior		0.074*
Only child or not		–0.045
Class and league cadres or not		–0.024
*df*	1144	1143
*R* ^2^	0.036	0.047
Δ*R*^2^	0.033	0.039
*F*	10.740***	5.597***

*P < 0.1, **P < 0.05, ***P < 0.001.

Table 4 shows the results of regression analysis on the factors that affect the behavior of female college students in China. As seen in Model 1*, while obedience motivation had no significant effect on the sports anomie behavior of female college students, academic motivation had a significant negative effect. To meet the requirements of sports attendance, pass the sports examination and obtain sports credits, stronger institutional motivation for female college students was needed because would be less likely to adopt anomie behavior (−0.137, *P* < 0.001). Economic motivation and sports anomie had a positive and significant impact. However, the stronger economic motivation of female college students to obtain scholarships and economic benefits meant that the possibility of adopting anomie behavior was higher (0.150, *P* < 0.001). Honor motivation and sports anomie had a negatively significant influence. The stronger the economic motivation of female college students to obtain scholarships and economic benefits, the less likely they were to adopt anomie behavior (−0.098, *P* < 0.1). Following Model 1*, Model 2* further introduced school grade, grade, whether the individual was an only child, and whether there were class and league cadres as control variables. Then, it was seen that the influence of academic motivation on the sports anomie of female college students was significant and that the direction of the regression coefficient did not change, but the regression coefficient itself decreased (−0.116, *P* < 0.001). The same thing was observed for economic motivation; however, the regression coefficient only slightly decreased (0.147, *P* < 0.001). What happened for economic motivation was also observed for honor motivation (−0.107, *P* < 0.1). Grade had a significant positive effect on the anomie behavior of female college students. As their grade increased, the anomie behavior of senior female college students also significantly increased (0.104, *P* < 0.1). Other control variables had no significant effect on the sports anomie behavior of female college students. Model 1 and Model 2 were significant; *R*^2^ increased with the increase of variables, which indicated that the model setting was effective.

**TABLE 4:** Multiple linear regression of sports anomie behavior of female college students.

Dependent variable: sports anomie	Model 1*	Model 2*
Independent variables		
Academic motivation	−0.137***	−0.116***
Obedience motivation	0.009	0.008
Economic motivation	0.150***	0.147***
Honor motivation	−0.098*	−0.107*
Control variables		
School level		–0.019
Sophomore		0.028
Junior		0.058
Senior		0.104**
Only child or not		–0.029
Class and league cadres or not		0.013
*df*	1194	1194
*R* ^2^	0.027	0.037
Δ*R*^2^	0.024	0.029
*F*	8.343***	4.508***

*P < 0.1, **P < 0.05, ***P < 0.001.

## Discussion

### Difference between sports anomie behavior and external motivation between male and female college students

This study found that the sports anomie behavior of female college students was significantly lower than that of the male students. First, from the analysis of the difference in the sports behavior between male and female students, it was seen that the difference in the sports behavior of male students was higher than that of female students and that the enthusiasm of male students to participate in vigorous sports was significantly higher than that of female students, making the probability of developing sports anomie higher (Zhu and Shu, 2022). Second, it was also related to the personality of the girls. Women are more docile with external regulations (Fleming and Agnew-Brune, 2015; Chen et al., 2017).

All the academic motivation scores of male and female students were above “strong,” and there was no gender difference. Foreign scholars believe that academic expectation and achievement factors are some of the causes of anxiety among college students (Armeli et al., 2001). Wang and Yao’s (2012) research has proved that academic pressure is the first source of pressure for contemporary college students, especially for those in China who have attached great importance to achievement and credit evaluation since childhood. Studies have shown that there is no gender difference in anxiety test among college students (Ang, 2022). In obedience motivation, economic motivation, and honor motivation, there were significant gender differences. The motivation of female students was weaker than that of male students. Ranking from the highest to the lowest, it was honor motivation, obedience motivation, and economic motivation. From the perspective of gender role analysis, a prescribed thinking mode was formed for expected behavioral norms among male and female students (Bern, 1975; Liu, 2011). Under traditional social culture, sports were generally considered as a symbol of solemnity and periodicity. It was considered as a display of the sports spirit of male students, while female students shouted and cheered for the men.

### Influence of external motivation on sports anomie behavior of male and female college students

There was a negative relationship between academic motivation and sports anomie behavior of male and female college students. Under oriented education and traditional Chinese culture, academic achievement was the most important pursuit of Chinese teenagers (Fuligni and Zhang, 2004). Students with strong academic motivation had higher academic goals. As such, they would not deter from their normal activities and develop anomie behavior for the sake of sports attendance, physical examination, credit, and other related systems. There was a positive relationship between the economic motivation and sports anomie behavior of male and female college students. Because female college students have lower physical fitness and physical exercise behavior than male students, they also have more economic motivation than male college students. Economic motivation referred to the desire to gain financial benefits through behavior in sports activities affected by adverse economic and social conditions. Under the background of the current era, college students are not ashamed to talk about “making money,” and when acknowledging the important role of money, they do not exaggerate its value (Hu, 2020). For example, to obtain scholarships, the hope was to help other students get rewards. This is narrated by students as such: “Students who agreed to help the test agent have good physical fitness, high test scores and generally have material benefits.” During the interviews, some students said: “She spent 100 Yuan to get the physical test done, and didn’t run at all. She also revealed to me that she was a student who was a middleman. Many students in our school contacted the runner through this student, and the student who was a middleman also made profits.” There was a negative relationship between the honor motivation and sports anomie behavior of female college students. A female college student who wanted to win in sports, gave credit to her team, and was praised by others must have had good physical and moral qualities. She must have had higher requirements for her personal code of conduct and less anomie.

In addition, *R*^2^ and the adjusted *R*^2^ of Model 1* and Model 2* were smaller than those of Model 1 and Model 2, respectively, which showed that the explanatory power of external motivation to the sports anomie behavior of male college students was greater, while the explanatory power to the sports anomie behavior of female college students was limited. This showed that there may have been more important variables that affected the sports anomie behavior of female college students.

### Other influencing factors of sports anomie behavior of male and female college students

Gender had a significant impact on the sports anomie behavior of college students, especially for boys. Existing literature showed that among the exercisers and non-exercisers, the scores of behavior habits, behavior intention and emotional experience of male college students were higher than that of their female counterparts, and the emphasis on gender was one of the important demographic factors (Wang, 2021). Multiple studies have shown that there are gender differences in adolescents’ attitudes and intentions to physical activity, and girls are more likely to have negative physical activity attitudes (Sun et al., 2018; Burton et al., 2020). In the interview materials of this study, there were many expressions about gender anomie, for example: “The most common anomie of sports in the teaching process is the lack of interest of students, especially the lack of attention of girls to sports, the phenomenon of asking for leave at will in class is more frequent, requiring probation or refusing to practice with a slight excuse.” “Boys are generally more likely to act impulsively, and may only win in the eyes of competition, losing will inevitably lead to anger, which will lead to quarrels or physical conflicts between the team or two teams. Different levels of schools have an impact on the anomie behavior of male college students”.

Studies have shown that the tolerance of students in 985 and 211 colleges and universities for behaviors involving illegal and moral bottom lines is significantly lower than that of vocational colleges (Wei, 2020). Students phrased it in the following ways: “We are higher vocational colleges, compared with students in undergraduate colleges, we are less conscious. The most common way of thinking in physical education is that we are lazy, do not want to make progress, and muddle along is particularly serious,” “Most vocational college students have poor self-study ability and less active practice,” “Junior college students and art students are relatively free in class,” “Private colleges and universities, most parents dote on their children,” and so on. The sports anomie behavior of the students in different grades was also different. The male students in junior and senior high school and the female students in senior high school all had significant sports anomie behavior. In the materials, it was stated as follows: “A large number of students are involved in a large number of sports classroom irregularities, involving a wide range of aspects and students of different grades.” “There are many students who take the place of the exam in the physical examination of the third and fourth grade of the undergraduate course just ended. As far as I know, most of these students take the place of the exam just because they don’t have physical education classes, they usually have no community activities to participate in, and they are lazy and don’t like activities very much. Every year, on the eve of college students’ physique test, students always complain a lot, and the physique test of the fourth year is the most headache. Because of the particularity of the fourth year, students in this grade are in a critical period from school to society. They are faced with the pressure of graduation design, postgraduate entrance examination, difficulty in employment, etc., and are prone to realistic problems of anxiety (Zhai et al., 2022). We can see the behavior of the fourth-year students kneeling in the circle of friends and asking for running for others, even paying for running for them to protect themselves, and there are not a few students willing to run for them.” Therefore, in the study of sports anomie, gender, different grades, and different schools were very critical factors.

### Limitations and prospects

Of course, there are some limitations to this research. First of all, as it is a cross-sectional research path, it is impossible to analyze the causality of the variables involved in the research. It is more scientific to use tracing research to further explore the mechanism of action between variables. Second, the college students’ sports anomie behavior and external motivation measurement scales are still not mature and complete. The scales of sports anomie behavior and exercise external motivation should be further supplemented and improved in follow-up research. Third, due to different regions, there are some differences in the implementation of school sports policies, which will have a certain impact on different college students’ sports behavior and external motivation. In the future, we will conduct more detailed exploration from the perspective of the region. Finally, the anomie behavior of sport is a complex concept because of the different types of sports, so it would be better to focus on one specific type of sports activities, due to multiple intervention strategies and studies.

## Conclusion and implications

Based on the previous research, several conclusions were drawn as follows: First, female college students’ sports anomie behavior is lower than that of male students. With the increase in grades, the sports anomie behavior of male and female students is on the rise. Second, the external motivation of female students is lower than that of male students. Third, academic motivation and economic motivation have a significant influence on college students’ sports anomie behavior.

The conclusion of this study has important implications for practice. First, physical education classes and extracurricular sports activities should be offered to juniors and seniors, especially to cultivate sports behavior as per norms. Second, physical education in ordinary schools should be examined specifically to strengthen the education of male college students’ anomie. Third, future research should target intervention studies to provide a theoretical reference for schools to better perform physical education. Research from the perspective of gender differences is conducive to targeted interventions on college students’ sports anomie behavior. The conclusions and suggestions drawn from the research provide theoretical support for health and education administrative departments to intervene in college students’ sports health behavior and health literacy and can improve school sports policies, the rationality of formulation, and the quality of sports management.

## Data availability statement

The raw data supporting the conclusions of this article will be made available by the authors, without undue reservation.

## Author contributions

LL and SC: formal analysis and writing—original draft preparation. XY: investigation, writing—review and editing, and supervision. All authors have read and agreed to the published version of the manuscript.
